# Getting Outside the Cell: Versatile Holin Strategies Used by Distinct Phages to Leave Their Bacillus thuringiensis Host

**DOI:** 10.1128/jvi.00696-22

**Published:** 2022-06-27

**Authors:** Audrey Leprince, Manon Nuytten, Elise July, Coralie Tesseur, Jacques Mahillon

**Affiliations:** a Laboratory of Food and Environmental Microbiology, Earth and Life Institute, Université Catholique de Louvain, Louvain-la-Neuve, Belgium; University of California, Irvine

**Keywords:** *Bacillus cereus* group, *Bacillus thuringiensis*, bacterial lysis, bacteriophages, endolysins, holin

## Abstract

Holins are small transmembrane proteins involved in the final stage of the lytic cycle of double-stranded DNA (dsDNA) phages. They cooperate with endolysins to achieve bacterial lysis, thereby releasing the phage progeny into the extracellular environment. Besides their role as membrane permeabilizers, allowing endolysin transfer and/or activation, holins also regulate the lysis timing. In this work, we provide functional characterization of the holins encoded by three phages targeting the Bacillus cereus group. The siphovirus Deep-Purple has a lysis cassette in which *holP30* and *holP33* encode two proteins displaying holin properties, including a transmembrane domain. The holin genes were expressed in Escherichia coli and induced bacterial lysis, with HolP30 being more toxic than HolP33. In Bacillus thuringiensis, the simultaneous expression of both holins was necessary to observe lysis, suggesting that they may interact to form functional pores. The myoviruses Deep-Blue and Vp4 both encode a single candidate holin (HolB and HolV, respectively) with two transmembrane domains, whose genes are not located near the endolysin genes. Their function as holin proteins was confirmed as their expression in E. coli impaired cell growth and viability. The HolV expression in B. thuringiensis also led to bacterial lysis, which was enhanced by coexpressing the holin with its cognate endolysin. Despite similar organizations and predicted topologies, truncated mutants of the HolB and HolV proteins showed different toxicity levels, suggesting that differences in amino acid composition influence their lysis properties.

**IMPORTANCE** The phage life cycle ends with the host cell lysis, thereby releasing new virions into the environment for the next round of bacterial infection. Nowadays, there is renewed interest in phages as biocontrol agents, primarily due to their ability to cause bacterial death through lysis. While endolysins, which mediate peptidoglycan degradation, have been fairly well described, the pore-forming proteins, referred to as holins, have been extensively characterized in only a few model phages, mainly infecting Gram-negative bacteria. In this work, we characterized the holins encoded by a siphovirus and two myoviruses targeting members of the Gram-positive Bacillus cereus group, which comprises closely related species, including the well-known Bacillus anthracis, B. cereus sensu stricto, and Bacillus thuringiensis. Overall, this paper provides the first experimental characterization of holins encoded by B. cereus phages and reveals versatile lysis mechanisms used by these phages.

## INTRODUCTION

Phages have evolved different mechanisms to release their virion progeny in the extracellular environment (1). For instance, filamentous phages extrude themselves without harming the host integrity, whereas single-stranded RNA (ssRNA) and single-stranded DNA (ssDNA) phages produce a single protein that inhibits the cell wall biosynthesis (2, 3). In contrast, double-stranded DNA (dsDNA) phages from the *Caudovirales* order (i.e., tailed phages) commonly harbor a lysis cassette encoding two types of lysis proteins, i.e., holin and endolysin (4). Their concerted action provokes bacterial lysis through inner membrane (IM) permeabilization due to holins, followed by endolysin degradation of the peptidoglycan (PG) meshwork.

Holins are small proteins, usually less than 150 amino acids (aa), possessing between one and four transmembrane domains (TMDs) and harboring a highly charged and hydrophilic C-terminal end (5). They have been classified into seven superfamilies and 52 families based on their topology and number of TMDs (6). Holins are responsible for the timing of lysis, which is paramount because premature lysis would lead to the release of incomplete phages, while delayed lysis could slow the infection of new hosts.

Phages belonging to the *Caudovirales* order have evolved two main pathways to achieve bacterial lysis. In the canonical pathway, lysis proteins are produced during the late gene expression phase, and fully active endolysins accumulate in the cytoplasm, while holins oligomerize harmlessly in the IM (7). At a particular moment called the triggering time, holins rearrange themselves to form microscale holes, leading to the collapse of the proton motive force (PMF) (8). These holes are large enough to allow endolysins to cross the IM and reach the periplasm, where they complete lysis by breaking down the PG (9, 10). Alternatively, in the noncanonical pathways, endolysins possess either a typical signal peptide (SP) or a signal-arrest-release (SAR) sequence that allows their direct translocation to the periplasm using a host secretion system (11, 12). However, they remain inactive in the periplasm, either free (SP endolysins) or tethered to the IM (SAR endolysins), until triggering time (13). At that moment, holins form numerous small holes, called pinholes, that allow the leakage of small ions, leading to the collapse of the PMF and the subsequent refolding and activation of the SP-/SAR endolysins (14).

In this work, we were interested in the holins encoded by distinct phages targeting members of the Bacillus cereus group. The taxonomy of this bacterial group is highly debated, and novel species are regularly proposed as new members (15). The seven best-known species of this group are B. cereus sensu stricto, Bacillus anthracis, Bacillus thuringiensis, Bacillus mycoides, Bacillus pseudomycoides, Bacillus weihenstephanensis, and Bacillus cytotoxicus. In contrast to endolysins encoded by B. cereus phages, which are well characterized (16–20), no data are yet available on their related holins. In this study, we focused on the characterization of holins encoded by the siphovirus Deep-Purple (21) and the myoviruses Deep-Blue (22) and Vp4, three phages infecting members of the B. cereus group.

## RESULTS

### *In silico* analysis identified putative holin candidates in phages Deep-Purple, Deep-Blue, and Vp4.

**FIG 1:**
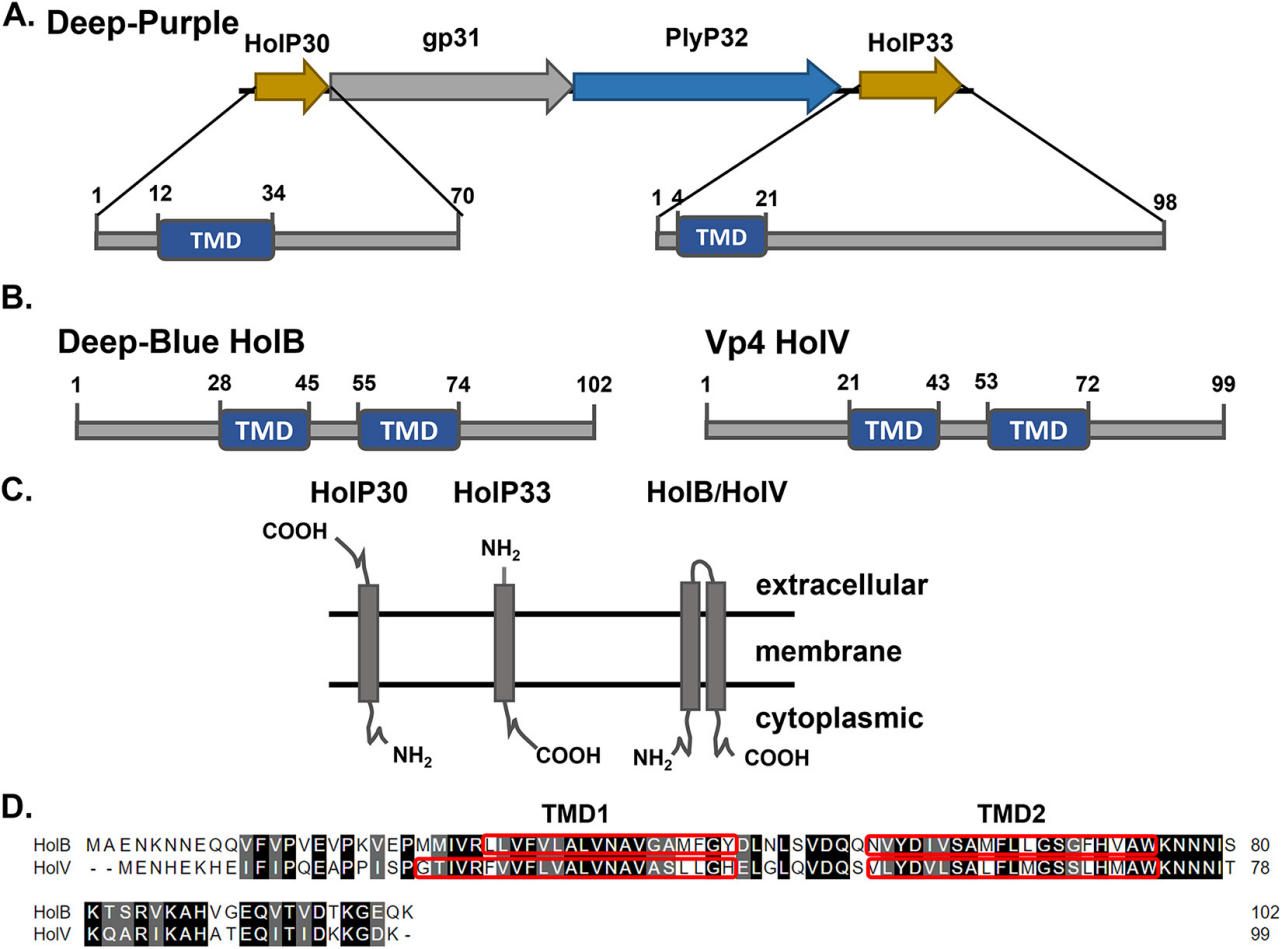
Holin general features. (A) In phage Deep-Purple, the lysis proteins are encoded in a lysis cassette comprising four genes, namely, *holP30* and *holP33*, encoding two holin-like proteins (yellow), *plyP32*, an endolysin (blue), and *gp31*, encoding a hypothetical protein (gray). Both putative holins have a single N-terminal TMD. (B) Deep-Blue (HolB) and Vp4 (HolV) candidate holins have similar organizations, with two central TMDs. (C) The potential topology of each putative holin is shown. (D) Sequence alignment between HolB and HolV is shown. TMDs are highlighted as red boxes and identical and similar amino acids with black and gray backgrounds, respectively. The numbers refer to the residue coordinates.

In phage Deep-Purple, the lysis cassette comprises four genes (i.e., *gp30* to *gp33*) (Fig. 1A). We recently characterized the endolysin PlyP32, which is encoded by *gp32* (20); *gp31* encodes a protein of unknown function, while *gp30* and *gp33* encode two proteins with holin features that were named HolP30 and HolP33, respectively. HolP30 is a 70-aa protein with an N-terminal TMD (residues 12 to 34) and a putative SP sequence with a cleavage site between amino acid 34 and amino acid 35. HolP30 has a predicted N-in/C-out topology and exhibits charged termini (Fig. 1C; also see Fig. S1 in the supplemental material). A BLASTp search showed that HolP30 has few matches with other putative holins, and no conserved domain could be identified. HolP33 is a 98-aa protein with a single TMD (residues 4 to 21), a short N-terminal extracellular portion, and a large cytoplasmic segment comprising several charged residues (Fig. 1A and C; also see Fig. S1). Similarly to HolP30, no conserved domain could be identified in the protein.

In phages Deep-Blue and Vp4, the putative holins HolB and HolV are encoded by *gp133* and *gp184*, respectively. In contrast to what is observed in many phages, these genes are not located in close proximity to their respective endolysin genes, *gp221* and *gp76*, respectively. HolB (102 aa) and HolV (99 aa) exhibit the same general protein organization, with two central TMDs and both termini presumably located in the cytoplasm (Fig. 1B and C). Search in the Transporter Classification Database (TCDB) assigned HolB and HolV to the SPP1 holin family (1.E.31), which consists of 90- to 160-aa proteins with two TMDs. Although the two proteins display similar arrangements, their sequences share only 51% identity, with the lowest level of conservation at their N-terminal ends (Fig. 1D).

### Expression of the four candidate holins induces cell lysis in Escherichia coli.

**FIG 2:**
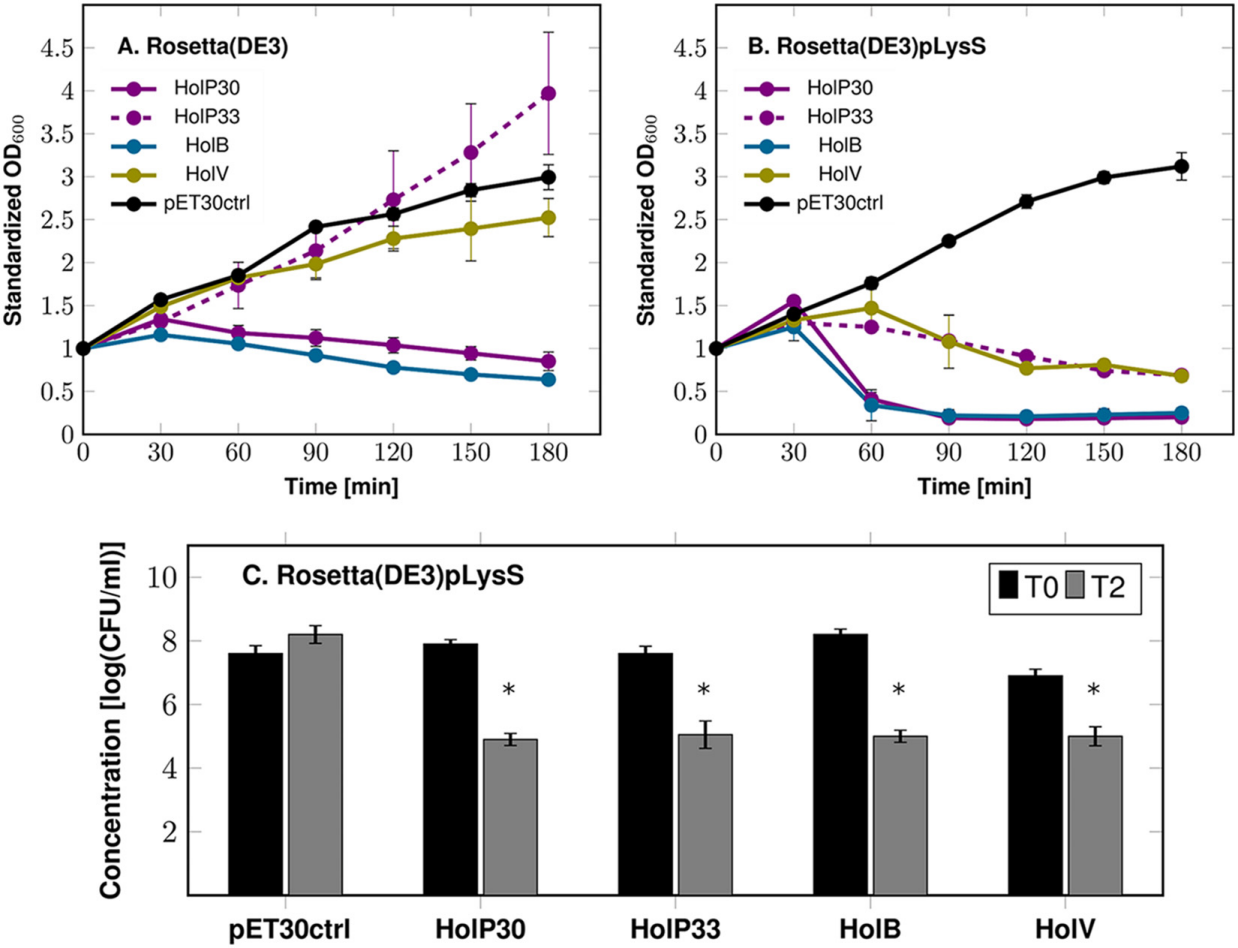
Expression of full-length holins in E. coli. The holins of Deep-Purple (HolP30 and HoP33), Deep-Blue (HolB), and Vp4 (HolV) were expressed in E. coli, and their effects on cell growth were assessed via OD_600_ monitoring and viable cell counting. Holin induction was done at time zero by adding 0.5 mM IPTG. (A) Monitoring of the OD_600_ upon IPTG induction for 3 h in the expression strain Rosetta(DE3). The data were standardized with respect to the OD_600_ at time zero. (B) Monitoring of the OD_600_ upon IPTG induction for 3 h in the expression strain Rosetta(DE3)pLysS, which expresses the T7 lysozyme. The data were normalized as in panel A. (C) Assessment of the viable counts before induction (T0) in Rosetta(DE3)pLysS and 2 h after IPTG induction (T2). The asterisks indicate statistically significant decreases of the CFU counts, compared to the noninduced conditions (T0). *, *P < *0.05 (Student’s *t* test). pET30ctrl represents the E. coli strain containing the empty expression vector. Standard deviations were derived from three independent experiments.

To assess the lytic activity of the full-length putative holins, *holP30*, *holP33*, *holB*, and *holV* were cloned into the vector pET30a and expressed in either E. coli Rosetta(DE3) or E. coli Rosetta(DE3)pLysS. The effect of the holin production on bacterial growth was measured by monitoring the optical density at 600 nm (OD_600_) upon isopropyl β-d-1-thiogalactopyranoside (IPTG) induction (Fig. 2A and B).

The two putative holins of Deep-Purple, HolP30 and HolP33, exhibited different behaviors upon expression. In Rosetta(DE3), the expression of HolP30 was toxic for the cells, as the density of the bacterial cultures expressing this holin gradually decreased with time (Fig. 2A). In contrast, the growth curve of Rosetta(DE3) expressing HolP33 was similar to that of the bacteria harboring the empty pET30 vector, showing that HolP33 was not lethal to the E. coli cells. In the Rosetta(DE3)pLysS background, HolP30 expression was even more toxic, as a rapid drop in the culture OD_600_ was observed after 60 min of induction (Fig. 2B). Interestingly, the expression of HolP33 also impaired cell growth, although to a lesser extent than what was observed for HolP30 (Fig. 2B).

Regarding the candidate holins of Deep-Blue (HolB) and Vp4 (HolV), although they displayed similar organizations (i.e., two central TMDs and the same predicted topology), they were able to induce cell lysis in E. coli to different degrees. In Rosetta(DE3), only HolB expression was lethal to the cells, while HolV had virtually no effect on the bacterial growth (Fig. 2A). Similar to what was observed for Deep-Purple holins, the bacterial lysis was quicker when HolB and HolV were expressed in Rosetta(DE3)pLysS, although HolV-mediated lysis remained moderate, compared to that observed for HolB (Fig. 2B).

The difference between the two expression strains used in this experiment is that Rosetta(DE3)pLysS expresses a T7 lysozyme that inhibits the T7 RNA polymerase, thus reducing the basal activity in pET vectors (23). In Rosetta(DE3), the sole expression of the holin led to moderate (HolP30 and HolB) or no (HolP33 and HolV) lysis, probably because it is linked only to the formation of pores in the IM, leading to cell content leakage and potential autolysin activation. In contrast, in Rosetta(DE3)pLysS, the T7 lysozyme released together with the cytoplasmic content can presumably act as an endolysin by breaking down PG, which allows rapid bacterial lysis. These observations suggested that the holes formed by the holins are large enough to allow the passage of macromolecular proteins such as the T7 lysozyme. The introduction of a stop codon in the T7 lysozyme gene of the pLysS plasmid confirmed the involvement of this enzyme in enhancing cell lysis (see Fig. S2 for details).

Reductions in culture OD_600_ values in Rosetta(DE3)pLysS expressing the holins were also linked to a decrease in counts of viable bacteria, as determined by assessing the bacterial concentrations before and after induction (Fig. 2C). Overall, a 3-log-unit reduction was observed after a 2-h induction period for HolP30 and HolB, while 2.5- and 2-log-unit drops were observed for HolP33 and HolV, respectively. Taken together, these results indicate that, in E. coli, all of the putative holins induce bacterial lysis and a reduction in cell viability, lending further support to the *in silico* predictions.

### The expression of holins alone is not sufficient to observe bacterial lysis in B. thuringiensis.

Since the holin candidates exhibited lytic activity when expressed in E. coli, we then aimed to evaluate their effect in AW43, a B. thuringiensis host for Deep-Purple, Deep-Blue, and Vp4 phages. The xylose-inducible shuttle vector pHT304pxyl, designed for protein expression in *Bacillus* (24), was used for monitoring the growth of AW43 expressing the different holins over a period of 24 h.

**FIG 3:**
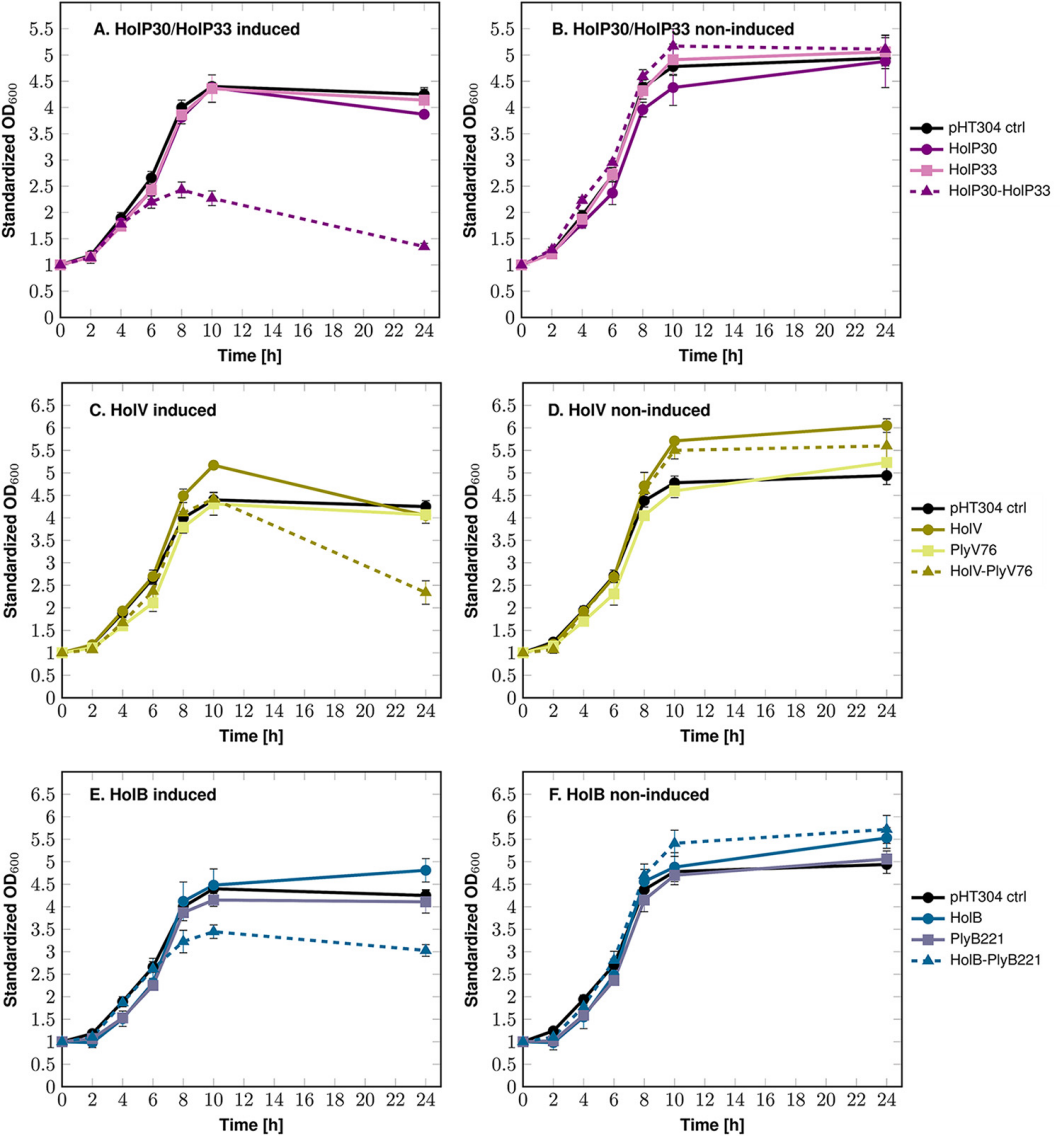
Expression of the full-length holins in B. thuringiensis AW43. The holins of Deep-Purple (HolP30 and HoP33), Deep-Blue (HolB), and Vp4 (HolV) were expressed in B. thuringiensis AW43, and their effects on cell growth were assessed via OD_600_ monitoring for 24 h. Holin induction was done at time zero by adding 20 mM xylose. For each graph, the data were standardized with respect to the OD_600_ at time zero. pHT304 ctrl represents the B. thuringiensis cells containing the empty expression vector. Standard deviations were derived from three independent experiments. (A) Individual expression of Deep-Purple holins (HolP30 and HolP33) and their coexpression (HolP30-HolP33). (B) Noninduced controls corresponding to Deep-Purple holins expression in panel A. (C) Expression of Vp4 holin (HolV) and endolysin (PlyV76) and their coexpression (HolV-PlyV76). (D) Noninduced controls corresponding to panel C. (E) Expression of Deep-Blue holin (HolB) and endolysin (PlyB221) and their coexpression (HolB-PlyB221). (F) Noninduced controls corresponding to panel E.

The Deep-Purple holins *holP30* and *holP33* were first expressed individually in AW43 (Fig. 3A and B). Surprisingly, over the course of induction, the growth curves of the cells expressing the holins were not impaired and were similar to that of the cells containing the empty expression vector. Given that the individual expression of HolP30 and HolP33 did not induce bacterial lysis as expected, we then evaluated the impact of the coexpression of the two holins. As shown in Fig. 3A, when AW43 expressed *holP30* and *holP33* simultaneously, a toxic effect was observed after 6 h of induction, as the cell growth began to slow. The highest OD_600_ value was reached 8 h after induction and was followed by a decrease in OD_600_ that led to an almost 2-fold drop after 24 h. Thus, the simultaneous production of HolP30 and HolP33 is necessary to induce cell lysis.

The holins of the myoviruses Deep-Blue and Vp4 were also expressed in B. thuringiensis AW43 (Fig. 3C to F). The cells expressing *holV* followed a normal growth curve during the first 10 h of induction, and a moderate diminution in OD_600_ (1.3-fold) was observed only at 24 h (Fig. 3C). As for the Deep-Blue holin HolB, no bacterial lysis was observed (Fig. 3E). Unlike Deep-Purple, in which two holins were identified, Deep-Blue and Vp4 encode only a unique obvious holin candidate; therefore, we wanted to test whether the coexpression of the holins and their respective endolysins would increase cell toxicity. For Vp4, the coexpression of *holV* with *plyV76* did enhance toxicity, as a decrease in the OD_600_ of almost 2-fold was measured (Fig. 3C). No cell lysis was observed when *plyV76* was expressed alone, highlighting the fact that the endolysin cannot induce cell toxicity on its own. As for Deep-Blue, the *holB*-*plyB221* coexpression only slowed the cell growth (Fig. 3E). Similar to what was observed in E. coli, HolB and HolV display distinct lysis behaviors. The correct holin expression was verified by Western blotting (data not shown).

### The holins seem to aggregate in the membrane.

**FIG 4:**
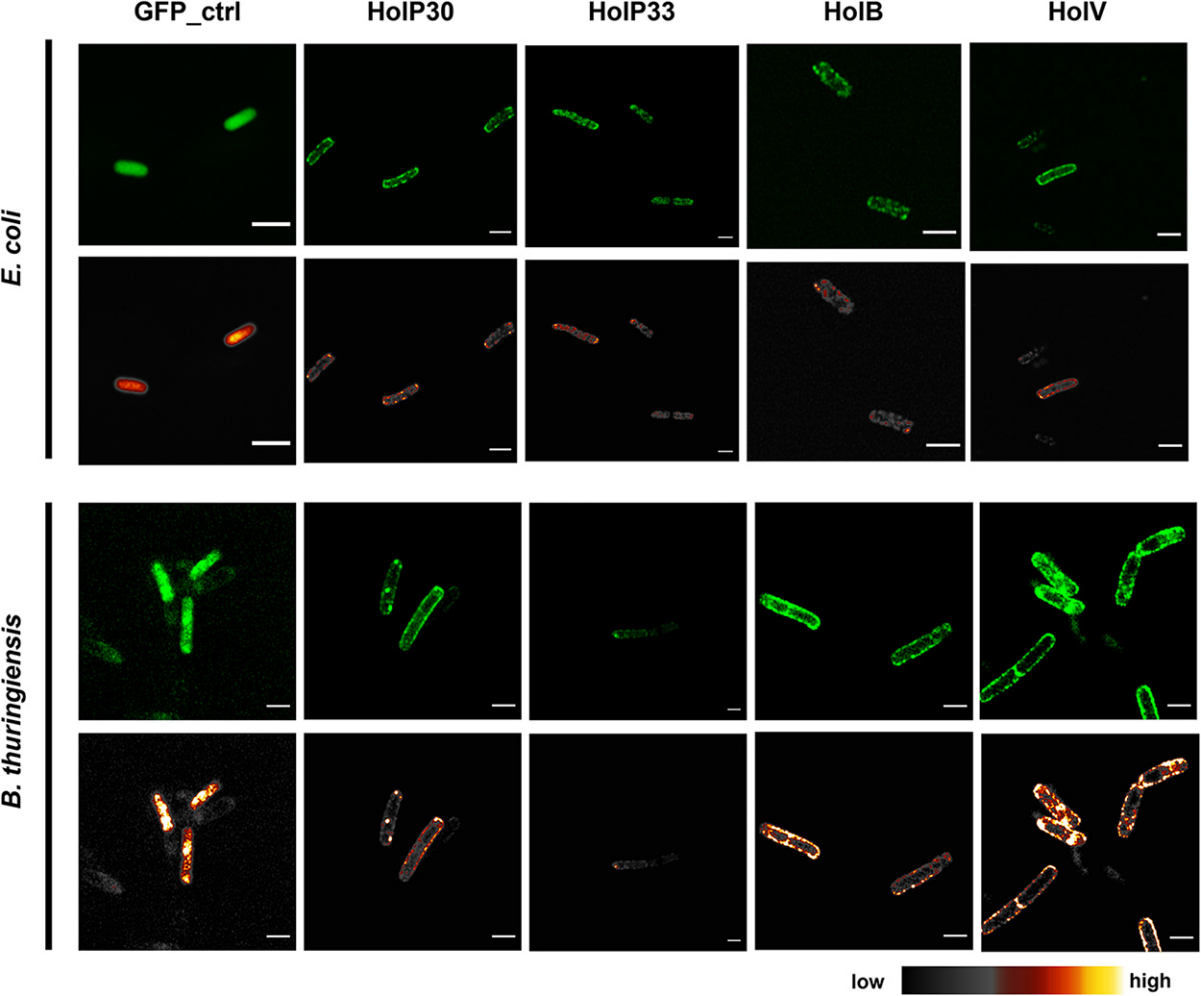
Confocal microscopic imaging of E. coli and B. thuringiensis cells expressing the full-length holins. The holins of Deep-Purple (HolP30 and HolP33), Deep-Blue (HolB), and Vp4 (HolV) were fused to a C-terminal GFP tag and expressed in E. coli BL21(DE3) using the expression vector pET30a and in B. thuringiensis AW43 using the xylose-inducible vector pHT304pxyl. GFP_ctrl represents bacteria expressing GFP alone. The upper rows show confocal microscopic images, while the bottom rows show corresponding images processed with ImageJ using a smart filter, indicating the fluorescence intensity (the scale is shown at the bottom). Scale bars represent 2 μm.

Next, we aimed to evaluate the holin localization in both E. coli and B. thuringiensis upon expression. To do so, we fused the four holins to a green fluorescent protein (GFP) tag in their C terminus and visualized them under the confocal microscope after 2 h of induction in either E. coli BL21(DE3) or B. thuringiensis AW43 (Fig. 4). Cells expressing the GFP alone were used as controls.

In E. coli, the holins localized at the cell periphery, presumably in the cell membrane, while the fluorescence associated with the GFP control was uniformly distributed throughout the cytoplasm (Fig. 4, upper rows). Interestingly, upon holin expression, the fluorescence was not uniform at the cell periphery. Instead, clusters of higher fluorescence intensity could be observed, especially in the case of HolP30, suggesting that the holins form aggregates in the cell membrane. Similar observations were made when the holins were expressed in B. thuringiensis (Fig. 4, lower rows).

### In Deep-Purple HolP30, both N- and C-terminal regions are necessary for cell toxicity.

**FIG 5:**
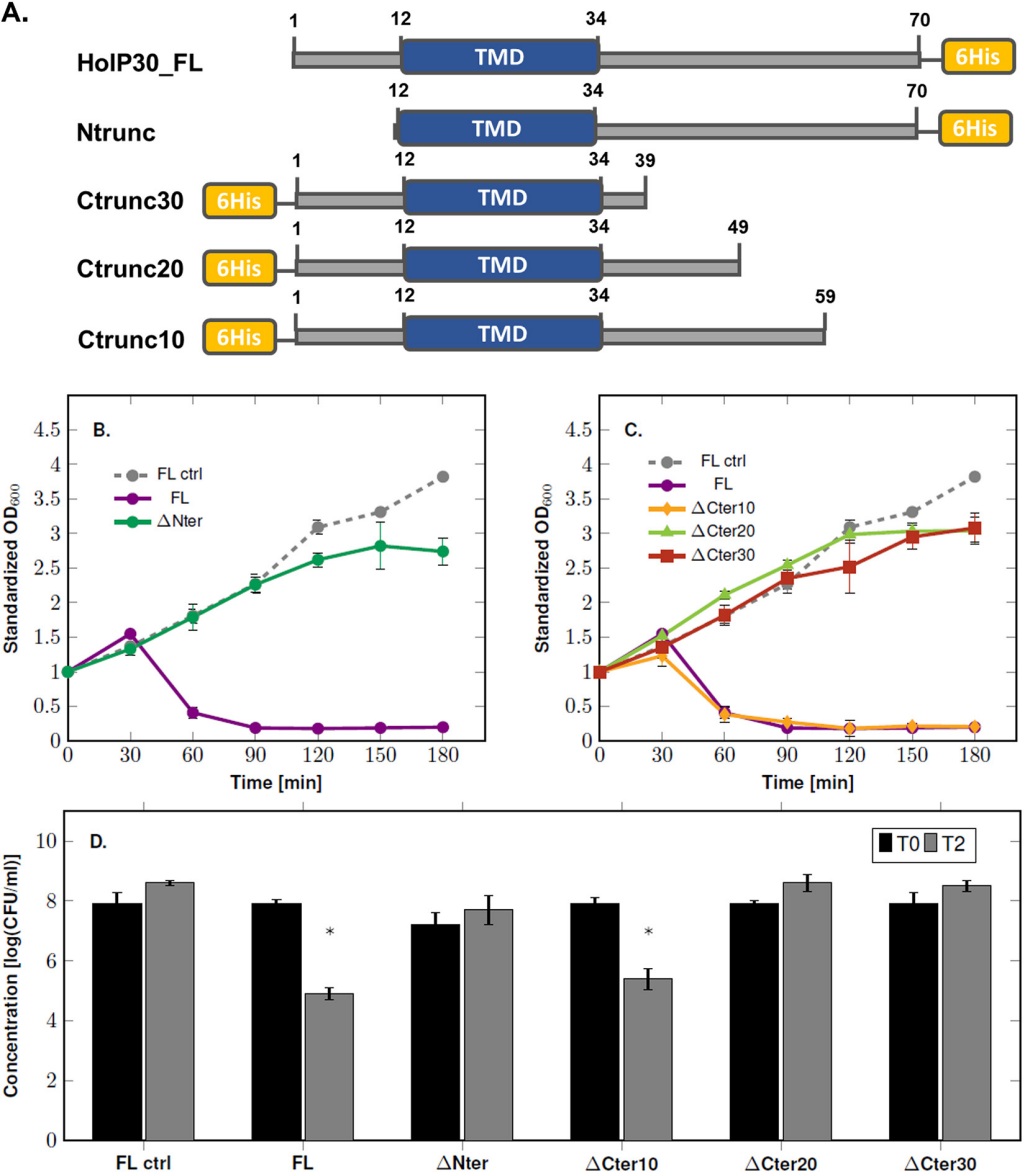
Expression of the truncated versions of the Deep-Purple holin HolP30. (A) Schematic representation of HolP30 truncated versions, including the location of the hexahistidine tag (yellow) and the TMDs (blue). The numbers refer to the residue coordinates. (B) Growth monitoring of E. coli Rosetta(DE3)pLysS expressing HolP30 truncated in its N-terminal part. The data were standardized with respect to the OD_600_ at time zero. (C) Growth monitoring of E. coli Rosetta(DE3)pLysS expressing three versions of HolP30 truncated in its C-terminal part by 10, 20, and 30 residues. The data were standardized as in panel C. (D) Assessment of the viable counts before IPTG induction (T0) in Rosetta(DE3)pLysS and 2 h after IPTG induction (T2). The asterisks indicate statistically significant decreases of the CFU counts, compared to the noninduced conditions (T0). *, *P < *0.05 (Student’s *t* test). Standard deviations were derived from three independent experiments. For comparison, the experiments performed with induced (FL) and noninduced (FL ctrl) full-length HolP30 are also shown.

In order to assess which part of HolP30 is involved in cell toxicity, we constructed truncated versions of HolP30 in which either its N terminus (ΔNter) or its C terminus (ΔCter) was removed (Fig. 5A). Moreover, for the C-terminally truncated HolP30, three different truncated versions were constructed, missing 10 (ΔCter10), 20 (ΔCter20), or 30 (ΔCter30) residues. The effect of the expression of each truncated HolP30 on E. coli Rosetta(DE3)pLysS was assessed by monitoring the culture OD_600_ and cell viability upon expression.

As shown in Fig. 5B, removal of the N-terminal domain abolished the cell toxicity, as the E. coli cells expressing HolP30ΔNter exhibited growth similar to that of the control corresponding to the noninduced bacteria containing the full-length HolP30. Similarly, no reduction in bacterial viability was observed for the E. coli cells expressing HolP30ΔNter (Fig. 5D). Regarding the C-terminally truncated HolP30, removal of the last 10 aa (HolP30ΔCter10) did not affect the protein toxicity, as the lytic effect was similar to that observed when the full-length HolP30 was expressed (Fig. 5C and D). Conversely, when HolP30 was truncated by at least 20 aa (HolP30ΔCter20 and HolP30ΔCter30), the bacteria had normal growth, and no toxic effect was observed (Fig. 5C and D). Thus, although the last 10 aa seem dispensable for HolP30 toxicity, the remaining part of the C-terminal domain appears to play an important role. The correct expressions of truncated holin versions were confirmed by Western blots performed with anti-6×His-tag antibodies (data not shown). Thus, it appears that, in HolP30, the presence of the TMD is not sufficient to induce cell toxicity but the N and C termini are necessary and may be the interacting domains involved in protein oligomerization.

### Despite sharing similar organizations, HolB and HolV display distinct cell toxicity levels.

**FIG 6:**
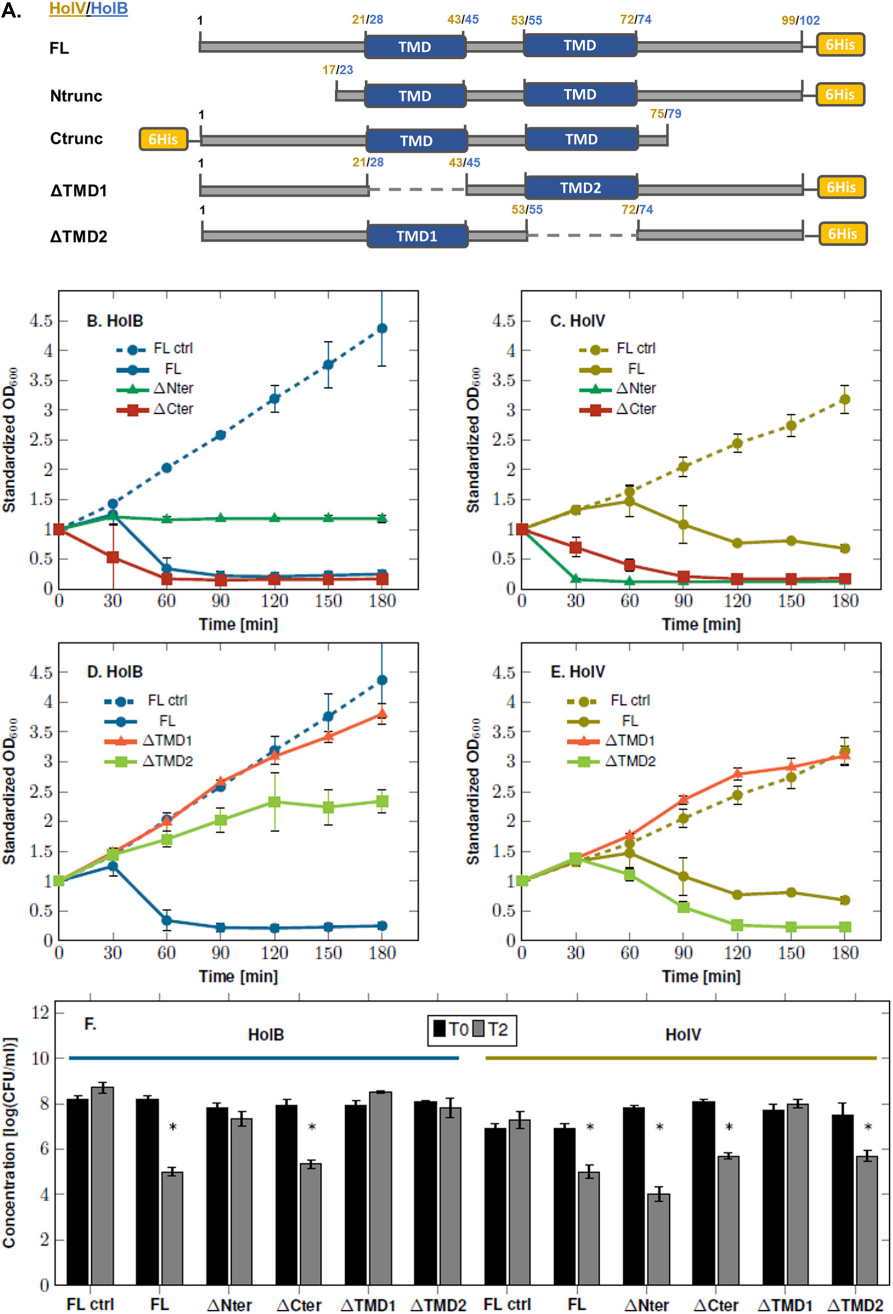
Expression of the truncated versions of HolB (Deep-Blue) and HolV (Vp4). (A) Schematic representation of the truncated versions of HolB and HolV. The two holins display similar organizations, with two central TMDs. The truncated versions correspond to holins without their N-terminal parts (Ntrunc), C-terminal parts (Ctrunc), or one of the TMDs (ΔTMD1 or ΔTMD2). In truncated versions without the TMDs, the missing TMDs are represented by a dashed line and both side of the holin-encoding gene were fused through Gibson assembly. The location of the hexahistidine tag (yellow) and the TMDs (blue) are indicated. The numbers refer to the residue coordinates of HolV in yellow and HolB in blue. (B and C) Growth monitoring of E. coli Rosetta(DE3)pLysS expressing N-terminally and C-terminally truncated versions of HolB (B) and HolV (C). The data were standardized with respect to the OD_600_ at time zero. (D and E) Growth monitoring of E. coli Rosetta(DE3)pLysS expressing HolB (D) or HolV (E) missing one of their TMDs. The data were normalized as in panels B and C. (F) Assessment of the viable counts before IPTG induction (T0) in Rosetta(DE3)pLysS and after 2 h (T2) for HolB and HolV derivatives. The asterisks indicate statistically significant decreases of the CFU counts, compared to the noninduced conditions (T0). *, *P < *0.05 (Student’s *t* test). Standard deviations were derived from three independent experiments. For comparison, the experiments performed with the induced (FL) and noninduced (FL ctrl) full-length holins are also shown.

Contrary to the Deep-Purple HolP30 and HolP33 holins, which have a single TMD, those of myoviruses Deep-Blue and Vp4 contain two TMDs. In order to assess the specific roles of these TMDs and their flanking sequences in protein toxicity, several *ad hoc* deletions were constructed (Fig. 6A). Their effects on cell growth and viability were then assessed in E. coli Rosetta(DE3)pLysS as in the case of Deep-Purple holins.

Removal of the C terminus had no impact on the ability of HolB to cause cell lysis, and the impact on cell viability remained unchanged, as an ~2-log-unit reduction was observed after 1 h of induction (Fig. 6B and F). In contrast, HolB truncated at its N terminus lost the ability to cause cell lysis, although the protein induction was still toxic to the cells, as an arrest in cell growth was observed (Fig. 6B). No significant diminution in cell viability after a 2-h induction of HolBΔNter was observed (Fig. 6F). For HolV, removal of either end of the protein had no impact on cell toxicity. In contrast, the decrease in OD_600_ was even more drastic than that observed for the full-length HolV (Fig. 6C). Regarding cell viability, the expression of HolVΔCter led to similar reductions of CFU counts, compared with the full-length HolV (i.e., 2.4 ± 0.2 and 1.9 ± 0.5 log units, respectively, after 2 h of induction), while the impact of HolVΔNter was more important, as a decrease in the CFU count of 3.8 ± 0.4 log units was observed after 2 h of induction (Fig. 6F).

To evaluate the involvement of the two TMDs in the toxicity of HolB and HolV, mutants lacking either the first or second TMD were constructed using the Gibson assembly method (Fig. 6A). In HolB, no bacterial lysis or decrease in cell viability was observed upon the expression of HolBΔTMD1 and HolBΔTMD2, highlighting that both TMDs are indispensable for HolB function (Fig. 6D and F). In HolV, however, removal of the first TMD completely abolished the holin lethal effect, while deleting the second TMD had no influence on HolV toxicity (Fig. 6E). This was further illustrated in the cell viability experiment, in which HolVΔTMD2 had a similar effect, compared with the full-length HolV, after 2 h of induction (i.e., 1.8 ± 0.7 and 1.9 ± 0.5 log units, respectively) (Fig. 6F).

## DISCUSSION

In contrast to holins encoded by phages targeting Gram-negative hosts, especially E. coli, those found in phages infecting Gram-positive bacteria have not been characterized in great detail (11, 25–27). Nonetheless, some Gram-positive lysis processes display interesting features that differ from what is commonly admitted for Gram-negative phages. For instance, in Streptococcus pneumoniae phage SV1, endolysins are thought to be cotransported with choline-containing teichoic acids in a holin-independent manner (28). In the present study, we characterized the holins encoded by Deep-Purple, Deep-Blue, and Vp4, three phages targeting members of the B. cereus group.

The siphovirus Deep-Purple has a lysis cassette comprising four genes, among which two encode putative proteins with holin features. HolP30 and HolP33 were predicted to belong to the class III holins, as they harbor a single TMD and differ mostly by their inverted predicted topology. Furthermore, HolP30 possesses a putative N-terminal SP, which was also found in the anti-holin RI of E. coli phage T4 (29). Interestingly, when expressed in E. coli, the lysis mediated by HolP30 was more drastic than that provoked by HolP33. Conversely, in B. thuringiensis, no cell toxicity was observed when the holins were expressed alone, despite their ability to insert in the membrane, as shown in localization experiments using fluorescence microscopy. Instead, the simultaneous expression of *holP30* and *holP33* was necessary to achieve bacterial lysis, suggesting that both proteins contribute to the holin function. This observation is reminiscent of what has been proposed for the B. subtilis siphophages SPP1 and PSBX, which also encode two proteins with holin properties within their lysis modules (30, 31). It is noteworthy that a two-holin system has also been described for the siphovirus Ms6, infecting Mycobacterium smegmatis (32). Further experiments are needed to establish how exactly HolP30 and HolP33 are involved in B. thuringiensis lysis and whether they interact with each other.

As for the myoviruses Deep-Blue and Vp4, only one putative holin, belonging to the class II holins (i.e., with two TMDs), was predicted. It is noteworthy that the corresponding holin genes do not cluster with their respective endolysin genes. A similar situation has already been described for the Streptomyces avermitilis phage phiSASD1 (25). The functional characterization of HolB and HolV showed that, although the two proteins display the same topology, they did not induce lysis to the same extent, as HolV-mediated lysis remained moderate, compared to that provoked by HolB. Similarly, the truncated holin mutants did not behave identically, suggesting that differences in amino acid composition and charge are likely to influence their lysis properties. In B. thuringiensis cells, the lethal effect linked to HolV expression was moderate, compared to what was previously observed for the simultaneous expression of Deep-Purple holins. Still, enhanced lysis was observed when HolV was expressed with its cognate endolysin, PlyV76, confirming a complementary role for the two proteins during lysis. In the case of Deep-Blue, the coexpression of HolB and PlyB221 only led to growth arrest. Localization experiments in B. thuringiensis combined with the demonstration of holin activities in E. coli strongly support a role for HolB and HolV in the lysis process. However, besides the holin and endolysins, other proteins may also be involved, and the simultaneous expression of all partners might be necessary to achieve an optimal and timely lysis. For instance, in the mycobacteriophage Ms6, two holin-like proteins are involved in the lysis timing, and a third protein with chaperone features is necessary for the endolysin export (32, 33). The fact that neither Deep-Blue nor Vp4 displays a lysis cassette makes difficult the identification of other potential partner proteins.

No SAR or SP endolysins have been described so far in B. cereus phages, suggesting holin-dependent export. This hypothesis was further supported in this work by comparing the holin induction in a classic E. coli strain with that in E. coli strains harboring a T7 lysozyme. Indeed, the lysis was enhanced in the presence of the T7 lysozyme, which strongly suggests that the membrane lesions formed by holins are large enough to allow the enzyme to cross the IM and reach the PG, thereby accelerating cell lysis.

In conclusion, this work provided novel insights into the holins involved in the phage-mediated lysis of Gram-positive hosts. Specifically, we showed that distinct phages infecting members of the B. cereus group use versatile mechanisms to achieve bacterial lysis before leaving their bacterial host.

## MATERIALS AND METHODS

### Bioinformatic analysis.

Conserved domains were identified using the Conserved Domain Database (CDD) (34) and the TCDB (35). Transmembrane helices were predicted using the TMHMM Server v. 2.0 (36).

### Bacterial strains, plasmids, and culture conditions.

Bacterial strains and plasmids used in this study can be found in Table 1. Bacteria were grown in lysogeny broth (LB) or on LB agar at 37°C for E. coli and at 30°C for B. thuringiensis unless stated otherwise. When necessary, media were supplemented with antibiotics (Sigma-Aldrich, Overijse, Belgium), i.e., 50 μg·mL^−1^ kanamycin (pET30a selection), 100 μg·mL^−1^ ampicillin (pHT304pxyl or pHT1618Kpxyl selection in E. coli), 200 μg·mL^−1^ kanamycin (pHT1618Kpxyl selection in B. thuringiensis), 10 μg·mL^−1^ erythromycin (pHT304pxyl selection in B. thuringiensis), or 10 μg·mL^−1^ chloramphenicol (E. coli Rosetta growth).

**TABLE 1:** Plasmids and strains used in this study

Strain or plasmid	Purpose^*a*^	Reference or source
Strains		
10-beta	E. coli cloning strain	NEB
C2925 (*dam*^−^/*dcm*^−^)	Methyltransferase-deficient E. coli	NEB
BL21(DE3)	T7 expression strain	Novagen
Rosetta(DE3)	T7 expression strain containing codons rarely used in E. coli	Novagen
Rosetta(DE3)pLysS	T7 expression strain containing codons rarely used in E. coli and pLysS plasmid expressing T7 lysozyme	Novagen
AW43	B. thuringiensis	38
Plasmids		
pET30a	E. coli expression vector	NEB
pHT304pxyl	E. coli/*Bacillus* shuttle and expression vector; xylose inducible	24
pHT1618Kpxyl	E. coli/*Bacillus* shuttle and expression vector; xylose inducible	39
pUC18::*gfp*	pUC18 vector containing GFP gene	Clontech/Takara
pAD43-25	CDS of GFP optimized for expression in *Bacillus*	BGSC
Deep-Purple HolP30 and HolP33 constructs		
pET30::*holP30*	Derivative of pET30 containing CDS of Deep-Purple full-length holin (HolP30) (amino acids 1–70) with C-terminal 6×His tag	This study
pET30::*holP30_Ntrunc*	Derivative of pET30 containing CDS of N-truncated version of HolP30 (amino acids 13–70) with C-terminal 6×His tag	This study
pET30::*holP30_Ctrunc10*	Derivative of pET30 containing CDS of C-truncated version of HolP30 (amino acids 1–59) with N-terminal 6×His tag	This study
pET30::*holP30_Ctrunc20*	Derivative of pET30 containing CDS of C-truncated version of HolP30 (amino acids 1–49) with N-terminal 6×His tag	This study
pET30::*holP30_Ctrunc30*	Derivative of pET30 containing CDS of C-truncated version of HolP30 (amino acids 1–39) with N-terminal 6×His tag	This study
pET30::*holP30*::*gfp*	Derivative of pET30 containing CDS of HolP30 with C-terminal GFP fusion	This study
pHT304pxyl::*holP30*::*gfp*	Derivative of pHT304pxyl containing HolP30 CDS with C-terminal GFP fusion	This study
pHT304pxyl::*holP30*	Derivative of pHT304pxyl containing HolP30 CDS with C-terminal 6×His tag	This study
pET30:: *holP33*	Derivative of pET30 containing HolP33 CDS with C-terminal 6×His tag	This study
pET30:: *holP33*::*gfp*	Derivative of pET30 containing HolP33 CDS with C-terminal GFP fusion	This study
pHT1618Kpxyl::*holP33*::*gfp*	Derivative of pHT1618Kpxyl containing HolP33 CDS with C-terminal GFP fusion	This study
pHT304pxyl::*holP33*	Derivative of pHT304pxyl containing HolP33 CDS with C-terminal 6×His tag	This study
pHT304pxyl::*holP30*::*rbs*::*holP33*	Derivative of pHT304pxyl containing HolP30 CDS and HolP33 CDS separated by RBS region	This study
Deep-Blue HolB constructs		
pET30::*holB*	Derivative of pET30 containing CDS of Deep-Blue full-length holin (HolB) (amino acids 1–102) with C-terminal 6×His tag	This study
pET30::*holB_Ntrunc*	Derivative of pET30 containing CDS of N-truncated version of HolB (amino acids 23–102) with C-terminal 6×His tag	This study
pET30::*holB_Ctrunc*	Derivative of pET30 containing CDS of C-truncated version of HolB (amino acids 1–79) with N-terminal 6×His tag	This study
pET30::*holBΔTMD1*	Derivative of pET30 containing HolB CDS without first TMD (amino acids 28–45) and with C-terminal 6×His tag	This study
pET30::*holBΔTMD2*	Derivative of pET30 containing HolB CDS without second TMD (amino acids 55–74) and with C-terminal 6×His tag	This study
pET30::*holB*::*gfp*	Derivative of pET30 containing HolB CDS with C-terminal GFP fusion	This study
pHT304pxyl::*holB*::*gfp*	Derivative of pHT304pxyl containing HolB CDS with C-terminal GFP fusion	This study
pHT304pxyl::*holB*	Derivative of pHT304pxyl containing HolB CDS with C-terminal 6×His tag	This study
pHT304pxyl::*plyB221*	Derivative of pHT304pxyl containing PlyB221 CDS	This study
pHT304pxyl::*holB*::*rbs*::*plyB221*	Derivative of pHT304pxyl containing HolB CDS and PlyB221 CDS separated by RBS region	This study
Vp4 HolV constructs		
pET30::*holV*	Derivative of pET30 containing CDS of Vp4 full-length holin (HolV) (amino acids 1–99) with C-terminal 6×His tag	This study
pET30::*holV_Ntrunc*	Derivative of pET30 containing CDS of N-truncated version of HolV (amino acids 17–99) with C-terminal 6×His tag	This study
pET30::*holV_Ctrunc*	Derivative of pET30 containing CDS of C-truncated version of HolV (amino acids 1–77) with N-terminal 6×His tag	This study
pET30::*holVΔTMD1*	Derivative of pET30 containing HolV CDS without first TMD (amino acids 21–43) and with C-terminal 6×His tag	This study
pET30::*holVΔTMD2*	Derivative of pET30 containing HolV CDS without second TMD (amino acids 53–72) and with C-terminal 6×His tag	This study
pET30::*holV*::*gfp*	Derivative of pET30 containing HolV CDS with C-terminal GFP fusion	This study
pHT304pxyl::*holV*::*gfp*	Derivative of pHT304pxyl containing HolV CDS with C-terminal GFP fusion	This study
pHT304pxyl::*holV*	Derivative of pHT304pxyl containing HolV CDS with C-terminal 6×His tag	This study
pHT304pxyl::*plyV76*	Derivative of pHT304pxyl containing PlyV76 CDS	This study
pHT304pxyl::*holV*::*rbs*::*plyV76*	Derivative of pHT304pxyl containing HolV CDS and PlyV76 CDS separated by RBS region	This study

a CDS, coding sequence.

### Plasmid constructions.

Plasmid constructs and primers used in this study are listed in Tables 1 and 2, respectively. PCR amplifications were performed using the Q5 high-fidelity DNA polymerase (New England Biolabs [NEB], Leiden, The Netherlands). Restriction enzymes were purchased from NEB and T4 DNA ligase from Promega (Leiden, The Netherlands). PCR and restriction products were purified using the GeneElute PCR clean-up kit (Sigma). Plasmids were transformed in E. coli 10-beta, and transformants were identified by PCR. Plasmids were extracted using the GeneElute plasmid miniprep kit (Sigma) and verified by sequencing (Macrogen, Amsterdam-Zuidoost, The Netherlands). Plasmid constructions were transformed into Rosetta(DE3) or Rosetta(DE3)pLysS for protein expression in E. coli. Prior to electroporation in B. thuringiensis AW43, plasmid DNA was demethylated by a passage in the E. coli *dam*^−^/*dcm*^−^ C2925 strain from NEB (37).

**TABLE 2:** Primers used in this study

Target and primer name	Primer sequence (5′→3′)^*a*^
Holins and truncated versions	
HolP30	
PHol70_NdeI_F	**ACTCATATG**ATTTCAAAAGAAGAACTACTA
PHol70_nostop_XhoI_R	**TATACTCGAG**TTCTCCTGTCTTATCTTCTT
HolP30_Ntrunc	
PHol58_NdeI_F	**TCTCATATG**AGTTGGCCTACTATT
PHol70_nostop_XHoI_R	**TATACTCGAG**TTCTCCTGTCTTATCTTCTT
HolP30_Ctrunc10	
PHol70_6His_NdeI_F	**TATCATATGCACCACCACCACCACCAC**TTGATTTCAAAAGAAGAACTACTA
PHol70_minus10_XhoI_R	**TATACTCGAGTTA**GTCATGCCAAATACCTAAAG
HolP30_Ctrunc20	
PHol70_6His_NdeI_F	**TATCATATGCACCACCACCACCACCAC**TTGATTTCAAAAGAAGAACTACTA
PHol70_minus20_XhoI_R	**TTTCTCGAGTTA**GGCGAATACATAAGGTAA
HolP30_Ctrunc30	
PHol70_6His_NdeI_F	**TATCATATGCACCACCACCACCACCAC**TTGATTTCAAAAGAAGAACTACTA
PHol70_minus30_XhoI_R	**ATTACTCGAG**TTAAAAGGTTTTCGTTTCTGC
HolB	
BHol102_NdeI_F	**CTCCATATG**GCAGAAAATAAAAACAATGAACA
BHol105_nostop_XhoI_R	**ATACTCGAG**TTTCTGTTCCCCTTTCGTAT
HolB_Ntrunc	
BHol80_NdeI_F	**TCCCATATG**ATGATTGTTAGACTATTAGTGTTC
BHol105_nostop_XhoI_R	**ATACTCGAG**TTTCTGTTCCCCTTTCGTAT
HolB_Ctrunc	
BHol102_6His_NdeI_F	**TATCATATGCACCACCACCACCACCAC**ATGGCAGAAAATAAAAACAATGAACA
BHol102-minus22_XhoI_R	**TATACTCGAG**TTAGATGTTGTTGTTCTTCCATG
HolV	
gp184_NdeI_F	**TATCATATG**ATGGAAAATCACGAAAAACACG
gp184_Nter_nostop_XhoI_R	**TATACTCGAG**TTTGTCTCCTTTTTTGTCGATTGTGAT
HolV_Ntrunc	
gp184_Nter_NdeI_F	**TATCATATGAT**GCCAATTTCCCCGGGTACTA
gp184_Nter_nostop_XhoI_R	**TATACTCGAG**TTTGTCTCCTTTTTTGTCGATTGTGAT
HolV_Ctrunc	
gp184_Cter_6his_NdeI_F	**TATCATATGCACCACCACCACCACCAC**GAAAATCACGAAAAACACGAAATATTCA
gp184-minus22_XhoI_R	**TATACTCGAG**GATGTTGTTGTTCTTCCACGC
HolP33	
gp33_NdeI_F	**TATCATATG**ACAATCGAGATAGGTTTATTATGT
gp33_XhoI_nostop_R	**TATACTCGAG**TTTAGCTTGTTTTTCCCGCATAAT
GFP fusions	
HolP30	
PHol70_NdeI_F	**ACTCATATG**ATTTCAAAAGAAGAACTACTA
gp30_EcoRI_R'	**AAAAGAATTC**TTCTCCTGTCTTATCTTCTT
HolB	
BHol102_NdeI_F	**CTCCATATG**GCAGAAAATAAAAACAATGAACA
gp133_EcoRI_R	**TATAGAATTC**TTTCTGTTCCCCTTTCGTATC
HolV	
gp184_NdeI_F	**TATCATATG**ATGGAAAATCACGAAAAACACG
gp184_EcoRI_R	**TATATGAATTC**TTTGTCTCCTTTTTTGTCGATTG
HolP33	
gp33_NdeI_F	**TATCATATG**ACAATCGAGATAGGTTTATTATGT
gp33_nostop_EcoRI_R	**ATATGAATTC**TTTAGCTTGTTTTTCCCGCATAAT
GFP E. coli	
GFP_EcoRI_linker_F	**TATAGAATTCGGTAGTGGATCAGGTAGTGGA**AAAGGAGAAGAACTTTTCACTGGAG
GFP_EagI_Stop_R	**TACGGCCGTTA**TTTGTAGAGCTCATCCATGCC
GFP *Bacillus*	
GFP_KpnI_linker_F	**AAGGTACCGGTAGTGGATCAGGTAGTGGA**AAAGGAGAAGAACTTTTCACTGGAG
GFP_stop_EcoRI_R	**TATAGAATTCTTA**TTTGTAGAGCTCATCCATGCC
GFP control *Bacillus*	
GFP_XbaI_F	**TTTCTAGA**AAAGGAGAAGAACTTTTCACTGGAG
GFP_stop_EcoRI_R	**TATAGAATTCTTA**TTTGTAGAGCTCATCCATGCC
Gibson primers for removal of TMDs	
HolVΔTMD1	
gp184TMD2_Cter_fwd	**AGTGGTGGTGGTGGTGGTGCTCGAG**TTTGTCTCCTTTTTTGTCG
gp184TMD2_Cter_rev	**CCAATTTCCCCG**GAGTTAGGTTTACAAGTAGAC
gp184Nter_fwd	**GTAAACCTAACTC**CGGGGAAATTGGCGGTGC
gp184Nter_rev	**ACTTTAAGAAGGAGATATACATATG**ATGGAAAATCACGAAAAACACGAAATATTCATCC
HolVΔTMD2	
gp184TMD2_Cter_fwd	**AGTGGTGGTGGTGGTGGTGCTCGAG**TTTGTCTCCTTTTTTGTCG
gp184_Cter_rev	**GTAGACCAAAGC**AAGAACAACAACATCACAAAAC
gp184TMD1_Nter_fwd	**TGTTGTTGTTCTT**GCTTTGGTCTACTTGTAAAC
gp184TMD1_Nter_rev	**ACTTTAAGAAGGAGATATACATATG**ATGGAAAATCACGAAAAAC
HolBΔTMD1	
BHol102Cter_fwd	**AGTGGTGGTGGTGGTGGTGCTCGAG**TTTCTGTTCCCCTTTCGTATC
BHol102TMD2_Cter_rev	**GATGATTGTTAGA**GACTTGAACTTATCAGTGG
BHol102Nter_fwd	**TAAGTTCAAGTC**TCTAACAATCATCATTGGTTC
BHol102Nter_rev	**ACTTTAAGAAGGAGATATACATATG**GCAGAAAATAAAAACAATGAAC
HolBΔTMD2	
BHol102Cter_fwd	**AGTGGTGGTGGTGGTGGTGCTCGAG**TTTCTGTTCCCC TTTCGTATC
BHol102Cter_rev	**AGTGGACCAGCAA**AAGAACAACAACATCTCGAAAAC
BHol102TMD1_Nter_fwd	**GTTGTTGTTCTT**TTGCTGGTCCACTGATAAG
BHol102TMD1_Nter_rev	**ACTTTAAGAAGGAGATATACATATG**GCAGAAAATAAAAACAATGAACAAC
pET30	
pET30_fdw	CATATGTATATCTCCTTCTTAAAGTTAAACAAA
pET30_rev	CTCGAGCACCACCACC
*Bacillus* expression	
HolP30	
PHol70_XbaI_F	**ATATCTAGA**TTGATTTCAAAAGAAGAACTACTA
PHol70-nostop-6his-KpnI_R	**TTGGTACCTTAGTGGTGGTGGTGGTGGTG**TTCTCCTGTCTTATCTTCTT
HolP33	
gp33_XbaI_F	**TTTCTAGA**ATGACAATCGAGATAGGTTTATTATGT
gp33_nostop_6His_KpnI_R	**TTGGTACCTTAGTGGTGGTGGTGGTGGTG**TTTAGCTTGTTTTTCCCGCATAAT
HolB	
BHol102_XbaI_F	**TATTCTAGA**ATGGCAGAAAATAAAAACAATGAACA
BHol102-nostop-6His-KpnI-R	**TTGGTACCTTAGTGGTGGTGGTGGTGGTG**TTTCTGTTCCCCTTTCGTATC
PlyB221	
gp221_XbaI_F	**TATTCTAGA**GCAATGTCTTTAGATACTTTAATCA
gp221_6His_stop_EcoRI_R	**TATTGAATTCTTAGTGGTGGTGGTGGTGGTG**CTCCTCAATGAAGTTGATGTATG
HolV	
gp184_XbaI_F	**TATTCTAGA**ATGGAAAATCACGAAAAACACG
gp184_nostop_6His_KpnI_R	**TTGGTACCTTAGTGGTGGTGGTGGTGGTG**TTTGTCTCCTTTTTTGTCGATTG
PlyV76	
gp76_BamHI_F	**ATAGGATCC**GCAATGGCATTACAAACTTTAATCG
gp76_nostop_6his_KpnI_R	**TTGGTACCTTAGTGGTGGTGGTGGTGGTG**TTTGAATGTACCCCAGTAATCTAC
Gibson primers for *Bacillus* coexpression	
pHT304pxyl	
pHT304pxyl_fwd	GTAATCATGTCATAGCTGTTTC
pHT304pxyl_rev	CATGTGATTTCCCCCTTAAA
HolB::RBS::PlyB221	
HolB_fwd	**TTTAAGGGGGAAATCACATG**GCAGAAAATAAAAACAATGAAC
HolB_rev	**ACATTGCCATACCACCGAGCCTCCTGAG**TTATTTCTGTTCCCCTTTC
PlyB221_fwd	**ACAGAAATAACTCAGGAGGCTCGGTGGT**ATGGCAATGTCTTTAGATAC
PlyB221_rev	**AACAGCTATGACATGATTAC**CTACTCCTCAATGAAGTTG
HolP30::RBS::HolP33	
HolP_fwd	**TTTAAGGGGGAAATCACATG**ATTTCAAAAGAAGAACTACTACG
HolP_revbis	**CGATTGTCATACCACCGAGCCTCCTGAG**TTATTCTCCTGTCTTATCTTC
gp33_fwd	**AGGAGAATAACTCAGGAGGCTCGGTGGT**ATGACAATCGAGATAGGTTTATTATG
gp33_rev	**AACAGCTATGACATGATTAC**TTATTTAGCTTGTTTTTCCCG
HolV::RBS::PlyV76	
HolV_fwd	**TTTAAGGGGGAAATCACATG**GAAAATCACGAAAAACACG
gp184_rev_bis	**TGCCATTGCCATACCACCGAGCCTCCTGAG**TTATTTGTCTCCTTTTTTGTC
gp76_fwd_bis	**GGAGACAAATAACTCAGGAGGCTCGGTGGT**ATGGCAATGGCATTACAAA
gp76_rev	**AACAGCTATGACATGATTAC**TTATTTGAATGTACCCCAGTAATC

a Primer extended ends are highlighted in bold.

Full-length holin genes (i.e., *holP*, *holB*, and *holV*) were cloned in pET30a using the restrictions sites NdeI and XhoI. The use of the NdeI restriction sites allowed removal of the purification tag region (i.e., S tag and 6×His tag). The holin stop codon was also removed to fuse it to the C-terminal 6×His tag present on pET30a.

The N-terminally truncated holin versions (i.e., *holP_Ntrunc*, *holB_Ntrunc*, and *holV_Ntrunc*) were cloned as for the full-length holins. The C-terminally truncated holin versions (i.e., *holP_Ctrunc*, *holB_Ctrunc*, and *holV_Ctrunc*) were also cloned using NdeI and XhoI restriction sites, but the 6×His tag was located at the N terminus and obtained with PCR amplifications of the truncated genes of interest using forward primers with a 6×His tag coding sequence (i.e., CACCACCACCACCACCAC) in the extended ends.

The deletions of either the first or second TMD in HolB and HolV (i.e., HolBΔTMD1, HolBΔTMD2, HolVΔTMD1, and HolVΔTMD2) were performed by first obtaining PCR amplicons with overlapping ends that missed one of the TMDs. The pET30a vector was linearized by PCR beginning from the vector start codon. Then, the two fragments and the linearized vector were fused using the HiFi DNA assembly method (NEB). The constructions harbor a C-terminal 6×His tag, which was added as indicated earlier for the C-terminally truncated derivatives.

For expression in B. thuringiensis using the pHT304pxyl vector, individual holin or endolysin genes were cloned by restriction/ligation. A C-terminal 6×His tag was added by PCR as indicated above. In order to perform coexpression experiments between the holins and their respective endolysins and between the two holins in the case of Deep-Purple, both genes were cloned in the pHT304pxyl vector (24) using the HiFi DNA assembly method (NEB). In this configuration, both genes maintained their native start and stop codons and were separated by a ribosome binding site (RBS) region (5′-CTC**AGGAGG**CTCGGTGGT-3′) (bold corresponds to the RBS sequence).

For fluorescence experiments, the full-length holin genes were cloned into pET30a (NdeI/EcoRI), and a GFP gene (*gfp*) was subcloned (EcoRI/EagI) in the C-terminal region. A short linker sequence (seven alternating glycine and serine residues) was inserted between the holin and the GFP tag. The same strategy was used for the pHT304pxyl or pHT1618Kpxyl cloning, except that the *gfp* gene used was amplified from pAD43-25 (Table 1), a vector that contains a *gfp* optimized for its expression in B. thuringiensis.

### Monitoring of bacterial cell growth upon holin induction in E. coli.

Overnight cultures of Rosetta(DE3) or Rosetta(DE3)pLysS carrying pET30a with holins or holin derivatives were used to inoculate fresh LB medium (1:25) and incubated at 37°C with agitation at 180 rpm until the OD_600_ reached 0.5. One-half of the culture was then induced with 0.5 mM IPTG (Sigma), whereas the other half served as the noninduced control. The cultures were further incubated at 30°C and 120 rpm, and the OD_600_ was monitored every 30 min over a 3-h period. Three independent experiments were performed, and the results were standardized with respect to the OD_600_ at the time of induction. The bacterial viability was assessed by collecting 1-mL samples (before induction and 2 h after induction), serially diluting the samples, and plating the dilutions on LB agar. The expression of the holins and their derivatives was verified by Western blotting using anti-His tag antibodies raised in mice (Bio-Rad, Temse, Belgium).

### Monitoring of bacterial cell growth upon holin induction in B. thuringiensis.

Overnight cultures of B. thuringiensis carrying pHT304pxyl with holins, endolysins, or coexpressions were used to inoculate fresh LB medium, and the OD_600_ was adjusted to 0.3. One-half of the culture was induced with 20 mM xylose (Sigma), whereas the other half served as the noninduced control. The cultures were further incubated at 30°C without agitation, and the OD_600_ was monitored over a 24-h period. Three independent experiments were performed, and the results were standardized with respect to the OD_600_ at the time of induction.

### Fluorescence microscopy experiments.

Strains containing plasmids with *gfp*-fused holin genes were expressed at a starting OD_600_ of 0.5 using 0.5 mM IPTG or 20 mM xylose for pET30a [transformed in E. coli BL21(DE3)] or pHT304pxyl (transformed in B. thuringiensis AW43), respectively. The cultures were incubated at 30°C for 2 h and observed using a confocal microscope (LSM710; Carl Zeiss).

### Data availability.

Holin sequences are available at NCBI GenBank with the following accession numbers: HolP30, YP_009833671.1; HolP33, YP_009833674.1; HolB, YP_009285445.1; HolV, ON527040; the PlyV sequence has the NCBI GenBank accession number ON527039.
