# Supplementary material for: Getting Outside the Cell: Versatile Holin Strategies Used by Distinct Phages to Leave Their Bacillus thuringiensis Host
Source: J Virol. 2022 Jun 27;96(14):e00696-22. doi: 10.1128/jvi.00696-22 (PMC9327680; doi:10.1128/jvi.00696-22)
Supplement: Supplemental file 1 — Fig. S1 and S2. Download jvi.00696-22-s0001.pdf, PDF file, 0.3 MB [file jvi.00696-22-s0001.pdf]

A. **Vp4 HolV (Gp184)**

MENHEKHEIFIPQEAPPISPGTIVRFVVFLVALVNAVASLLGHELGLQVDQSVLYDVLSALFLMGSSLHMAWKNN
NITKQARIKAHATEQITIDKKGDK

Vp4 PlyV76 (Gp76)

MAMALQTLIDKANRKLNVSGMRKDVADRTRAVITQMHAQGIYICVAQGFRSFAEQDALYAQGRTKPGSIVTNARG
GQSNHNYGVAVDLCLYTQDGSDVIWTVEGNFRKVIAAMKGQGFKWGGDWVSFKDYPHFELYDVVGGQKPPADNGG
AVDNGGGSGGSSGGSTGGGSTGGDYDSSWFTKETGTFTTNTAIKLRTSPFTSAGVIATLPAGSVVNYNGYGIEYN
GYVWIRQPRSNGYGYLATGESRNGKRVDYWGTFK

B. **HolP30 (Gp30)**

+-- ++ - + -+ - - +-- -

MISKEELLRRL**MSWPTIVAIVSLIGFLATTAGHA**ETKTFLDKLLPYVFAVGTALGIWHDHEPIKEDKTGE

HolP33 (Gp33)

- + +-- ++ - + - + - + - +

MTI**EIGLLCVIVGAIVSAVGL**KMTLQKNTKEEARKQAEMSVKLDSMANGINNIQIKIEAQNNKLEAFNVR

+ -- + + - +-+ +

LVRVEESTKSLHNRVNSVESIMREKQAK

HolB (Gp133)

- + - - + - + - - -

MAENKNNEQQVFVPVEVPKVEPMMIVR**LLVFVLALVNAVGAMFGY**DLNLSVDQQN**VYDIVSAMFLLGSGFHVAWK**N

+ + + - - + - +

NNISKTSRVKAHVGEQVTVDTKGEQK

HolV (Gp184)

- -+ - + - - - +

MENHEKHEIFIPQEAPPISP**GTIVRFVVFLVALVNAVASLLGH**ELGLQVDQS**VLYDVLSALFLMGSSLHMAW**KNNN

+ + + - -++ -+

ITKQARIKAHATEQITIDKKGDK

Figure S1 – Sequence analysis of holin proteins. A. Protein sequence of Vp4 holin (HolV) and endolysin (PlyV76). **B.** Negatively (-) or positively (+) charged amino acids are indicated in the protein sequences of the putative holins of Deep-Purple (HolP30 and HolP33), Deep-Blue (HolB) and Vp4 (HolV). The predicted transmembrane domains are highlighted in bold.

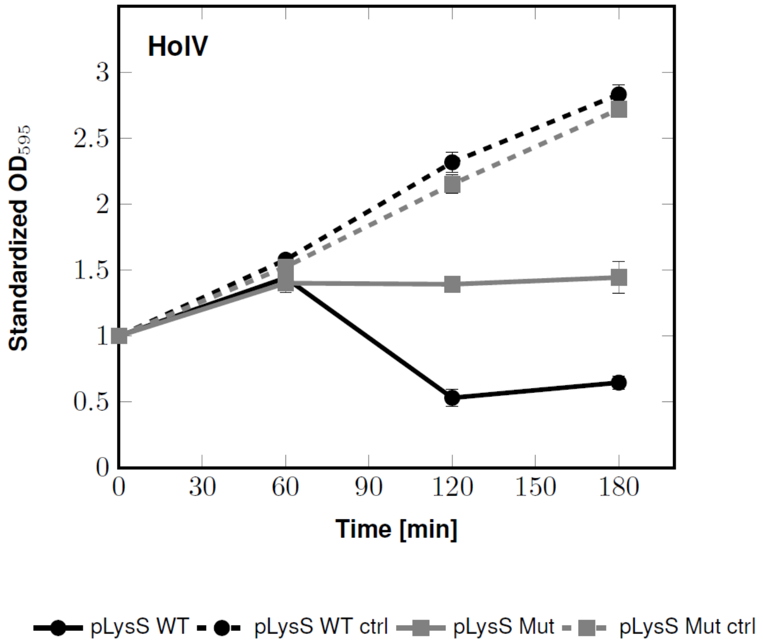

Figure S2 – Comparison of the expression of HolV in BL21(DE3) containing either the wildtype pLysS plasmid (pLysS WT) or a mutated version (pLysS Mut). An amber codon was placed in the T7 lysozyme gene using the Q5 site-directed mutagenesis kit from NEB. Then the wildtype and mutant plasmids were transformed into BL21(DE3) as well as pET30 containing the HolV gene. The effect of HolV on cell growth was assessed via OD_{600} monitoring where induction was done at t=0 min by adding 0.5 mM of IPTG. The data were standardized with respect to the OD_{600} at time 0. “ctrl” indicates non-induced conditions.
